# Inventory reveals wide biodiversity of edible insects in the Eastern Democratic Republic of Congo

**DOI:** 10.1038/s41598-022-05607-y

**Published:** 2022-01-28

**Authors:** Jackson Ishara, Rodrigue Ayagirwe, Katcho Karume, Gustave N. Mushagalusa, David Bugeme, Saliou Niassy, Patchimaporn Udomkun, John Kinyuru

**Affiliations:** 1 Department of Food Science and Technology, Université Evangélique en Afrique, P.O. Box 3323, Bukavu, Democratic Republic of Congo; 2 Department of Food Science and Technology, Jomo Kenyatta University of Agriculture and Technology, P.O. Box 62000-00200, Nairobi, Kenya; 3 Department of Animal Sciences, Université Evangélique en Afrique, P.O. Box: 3323, Bukavu, Democratic Republic of Congo; 4 Department of Crop Sciences, Université Evangélique en Afrique, P.O. Box: 3323, Bukavu, Democratic Republic of Congo; 5 Crop Production and Protection Unit, Université Catholique de Bukavu, Bukavu, Democratic Republic of Congo; 6 International Centre of Insect Physiology and Ecology (Icipe), P.O. Box 30772-00100, Nairobi, Kenya; 7 International Institute of Tropical Agriculture (IITA), Bujumbura, Burundi

**Keywords:** Animal behaviour, Entomology

## Abstract

In response to growing food demand, edible insects are perceived as an opportunity to alleviate food insecurity. With its wide edible insects’ biodiversity, the Democratic Republic of Congo is one of Africa’s most critical entomophagous. This study aimed at giving a first insight on inventory showing diversity, perception, consumption, availability, host plants, harvesting techniques and processing techniques of edible insects in South-Kivu, DRC. It recorded twenty-three edible insects belonging to nine families and five orders, some of which are consumed in the larval, adult, egg and pupa stages. *Rhyncophorus phoenicis*, *Alphitobius diaperinus*, *Macrotermes subhyalinus* and *Acheta domesticus* were the most preferred edible insects in Fizi Territory, *Ruspolia differens* and *Apis mellifera* larvae in Kabare Territory, *Imbrasia oyemensis*, *Imbrasia epimethea*, *Rhynchophorus ferrugineus* and *Rhyncophorus phoenicis* in Mwenga Territory, *Ruspolia differens*, *Macrotermes subhyalinus*, *Gryllotalpa africana*, Nsike, *Nomadacris septemfasciata* and *A. mellifera* larvae in Walungu Territory. *Ruspolia differens*, *I. oyemensis*, *A. mellifera* larvae, *G. africana* and Nsike, were preferred for their taste. *Acheta domesticus*, *A. diaperinus* and *A. mellifera* larvae were abundant throughout the year, while others were only available for 9 months or less per year. Numerous plants have been recorded as their hosts, including plants used for food and income. Harvesting strategies and period, processing methods and preservation techniques depend on insect species, local knowledge and practices. These findings suggest similar and thorough studies on entomophagy across the country while encouraging the rearing of edible insects to address their existing high demand and environmental concerns.

## Introduction

The world's population is expected to reach 9.8 billion by 2050 and 11.2 billion by 2100^1^, raising concerns about food production and the ever-growing demand for protein^2^. Edible insects are among the most important bioresources being promoted to address global food and nutritional security^3–5^. Worldwide edible insects are regularly consumed by 2 billion people^6,7^ for their nutritional value and taste^8,9^. Several studies have demonstrated the superior nutritional value of edible insects compared to conventional foods^10–13^.

The most consumed groups of edible insects include beetles (Coleoptera, 31%), caterpillars (Lepidoptera, 18%), and bees, wasps, and ants (Hymenoptera, 14%), followed by grasshoppers, locusts and crickets (Orthoptera, 13%), cicadas, leafhoppers, planthoppers, scale insects and true bugs (Hemiptera, 10%), termites (Isoptera, 3%), dragonflies (Odonata, 3%), flies (Diptera, 2%) and 5% other orders^13^. The availability of some edible insects depends on the geographical distribution of their host plants and seasonality^14^ and correlates with their harvesting period^15^. In turn, successful edible insect harvest depends on insect habits and ecological factors^16^, as they are harvested at different growth stages, including the larval (bees, beetles, butterflies, and ants) and adult (beetles, ants, grasshoppers) stages. For some species, harvesting is easy at night or early in the morning when inactive and cannot fly^17^.

In Africa, harvesting techniques of edible insects are a mixture of observations, hand-picking, tracings, sign interpretations, and trapping strategies^18^, varying from one insect to another. The most commonly used harvest techniques are hand-picking, mainly for crickets, Rhinoceros beetle, African palm weevil, and caterpillars, while light trapping is the most used technique to harvest termites, green grasshoppers, house cricket, and mole cricket^19,20^.

Studies on edible insects have been conducted in Africa, America, Asia, Europe and Australia to assess key variables in entomophagy perception and practices^5,13,16,21–27^. Like many African countries, food security has been a significant challenge in the Democratic Republic of Congo (DRC), exacerbated by climate change^28^, forcing disadvantaged communities to desperately depend on forest products, including edible insects, as they are among the most abundant forest resources^29,30^. They play an incredible role throughout the year, especially during the rainy season, depending on the geographical location of different cultures^31^.

Despite the vast diversity of edible insects in the DRC, there are few studies on edible insects’ inventory except the study conducted on ecological diversity of edible insects and their potential contribution to household food security in Haut-Katanga Province^31^ and in the area surrounding LuiKotale, Salonga National Park^32^. However, to the best of our knowledge, there is little-to-no research on the biodiversity, perception, consumption, availability, host plants, harvesting techniques, and processing techniques of edible insects in South Kivu. Studies from other regions cannot be extrapolated to South-Kivu consumers due to ecological, cultural and dietary habit differences, meaning there is a need for more detailed study in this regard.

## Material and methods

### Ethics statement

All experimental protocols, as well as methods, were approved and carried out as per relevant guidelines and regulations from the Interdisciplinary Centre for Ethical Research (CIRE) established by the Evangelical University in Africa, Bukavu, D.R. Congo, with reference (UEA/SGAC/KM 132/2016). All subjects signed an informed consent form describing the aim of the study after translation into local languages.

### Study area

**Figure 1:**
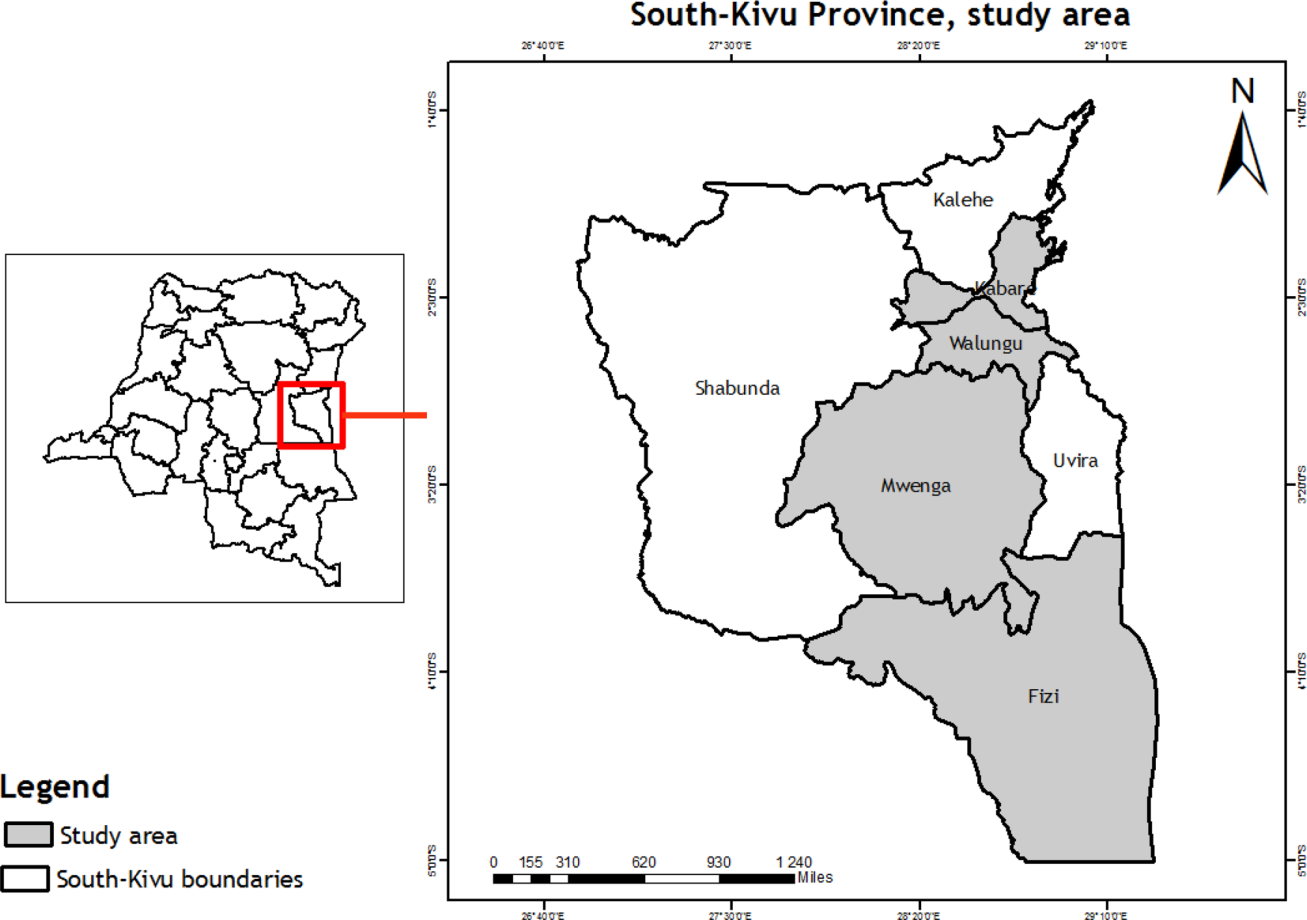
Map showing South-Kivu Province and the study Territories (ArcMap 10.4. https://desktop.arcgis.com/en/arcmap/10.4/).

The survey and direct observation were carried out in four selected Territories (Fizi, Kabare, Mwenga and Walungu) of South-Kivu Province, D.R. Congo (Fig. 1), where data on diversity, host plants, seasonal availability, harvesting techniques and traditional processing, consumption, preference of edible insects were collected. These Territories were purposively selected for their familiarity with entomophagy as local communities traditionally practice it. Territories are in different agroecological conditions with different cultures influencing edible insects’ availability and consumption.

### Agro-ecological conditions of the study area

The agro-ecological conditions of the study area are presented in Table 1. The Fizi Territory is located between 3° 30 and 4° 51 32 latitude (South), and 27° 45 and 29° 14 10 longitude (East). Its elevation is subdivided into four zones, including the coastline (~ 750 m), the low land valley (~ 1000 m), a highland (~ 1300 m), and the very highland (locally called Haut Plateau with 1700 m). The climate in Fizi is highly affected by the elevation. The rainfalls are unevenly distributed according to the month and the climatic subdivision. The North, dominated by the coastline and low inland valley, is characterised by humid tropical climate (of Aw3 type according to Köppen–Geiger classification). The greatest rainfall amounts are recorded in March and November, while the smallest amounts are the smallest amounts in February and September. The south part has a dry humid tropical climate. Available climate data mentioned an average annual rainfall of ~ 1704 mm, the mean temperature ~ 23.54 °C (with the highest observed in April with ~ 25.6 °C and the lowest ~ 21.3 °C in September). The Territory is dominated by forest, comprising two forest reserves and a nature reserve. Acrisols and Cambisols are the dominant soil unities according to the WRB classification.

**Table 1:** Agro-ecological conditions of the study area (retrieved from CAID).

Characteristics	Territory			
	Fizi	Walungu	Kabare	Mwenga
Latitude (South)	3° 30 to 4° 51′ 32	2° 38′	2° 30′	3° to 4°
Longitude (East)	27° 45 to 29° 14′ 10	28° 40′	28° 30′	28° 25′ 29″
Area (km^2^)	15,789	1800	1960	11,172
Altitude (m)	750 to 1700	1000 to 2000	1420 to 3200	670 to 1800
Climate type	Humid wet and dry tropical	Humid wet tropical	Humid wet tropical	Equatorial
Dominant soil unity	Acrisols and Cambisols	Ferralsols, Cambisols and Nitisols	Ferralsols and Nitisols	Acrisols and Cambisols
Mean T °C	23.54 °C	17–20 °C	22.6 °C	21–37 °C
Mean annual P (mm)	1704	900 to 1500	1572	1650
Estimated population (2019)	1,093,926	1,509,175	868,616	843,636
Density of population (hab km^−2^)	69.3	838.4	443.6	75.5
AEZ*	Low and high altitude	Medium to high altitude	Medium to high altitude	Low and high altitude

*P (mm)* Precipitation (rainfall), *AEZ* Agro-Ecological Zone (High, Medium, Low).

The Kabare Territory is located between 2° 30′ of South latitude and 28° 30′ of East longitude. Its altitude varies from ~ 1420 to 3200 m, and the Territory occupies an area of ~ 1690 km^2^ with an estimated population of ~ 868,616, which makes it among the most populated in the South-Kivu province. The Territory is located in the medium to high altitude AEZ. Available meteorological data mentioned an annual rainfall average of ~ 1572 mm, and a temperature of ~ 22.6 °C. Most of Kabare is savanna with natural vegetation consisting of wild grasses.

The Mwenga Territory is located in the middle of the province and is the only Territory surrounded by the other without any country or province borders. It is located between 28° 25′ 29″ East longitude and 30° 02,16′ 05″ South latitude. Its altitude varies between 1500 and 1800 m in the northeast. In the centre and the South, it is more or less 670 m. In the East, it is more or less 200 m and in the West more or less 670 m. It has a humid tropical climate with two seasons: the dry season from June to September and the rainy season from September to May. The temperature varies between 21 and 37 °C in most of the Territory and is low in the Itombwe area because of the high altitude, which goes up to over 2000 m. Rainfall reaches 2000 mm to 3000 mm per year. The vegetation is mainly dense forest and savanna. The forest is home to the Itombwe Nature Reserve (RNI). Relief is dominated by the Itombwe mount uplands and the alluvial valley of the Elila watershed. Soils dominated with clayey (Humic Cambisols) and sandy soil (Acrisols) types.

The Walungu Territory is located between 2° 38′ of South latitude and 28° 40′ of East longitude. Its altitude varies between 1000 m and 2000 m with a cold tropical climate of low altitude. There are two seasons, the dry season (June to August) and the rainy season from September to March. Available station data presented an annual average of ~ 17–20 °C and 900 and 1500 mm for temperature and rainfall, respectively. The vegetation mainly consists of grassland, a few forest reserves of Mugaba and Mushwere and woodlands scattered throughout the Territory.

### Sampling and selection of respondents

A total of 520 respondents, about 130 respondents in each Territory, were interviewed, with priority given to people familiar with entomophagy based on the main objectives set. Therefore, the respondents included adults, women and men over 18 years old and from all social classes. A structured oral interview was used individually to ensure better information and minimise external influences on the respondent's side.

### Sources of data collected

Primary data were obtained from the field survey using three techniques: questionnaire administration, direct observations, and insect collection.

#### Questionnaire administration

Structured questionnaires were used to obtain information on edible insects in all selected Territories of South-Kivu. The questionnaire was divided into seven sections. In the first section, information about the respondents was collected. Section two contained open-ended questions related to commonly consumed edible insects, focusing on local names and stages of consumption. The third section contained questions about consumer preferences and preference factors. The fourth section included questions related to seasonal availability. In the fifth section, questions about host plants and signs of presence were asked, followed by personal observations. The sixth section dealt with harvesting techniques and timing. The last section dealt with processing methods and preservation techniques. The enumerators translated the questions into the local dialect to enhance the understanding of respondents. Pictures and real samples of various edible insects identified from the literature were also used to help respondents identify the insects being mentioned. Enumerators probed further to clarify some responses to enhance the depth of information solicited.

#### Direct observations

Direct observations of relevant information related to insects and their habitats in the different territories were recorded in the field. Pictures were taken to verify and support the responses obtained from the interviewees. In addition, the researcher had the opportunity to observe how some edible insects were prepared and consumed.

#### Collection and taxonomic identification of insect samples

Samples of edible insects were collected as part of the survey and were preserved in 70% alcohol before being taken to the laboratory at Lwiro Research Center for identification. A mixture of primary data and taxonomic characters was used to identify and classify the various species of edible insects in the different Territories. The taxonomic characters were derived mainly from archival sources and published literature.

### Data analysis

Data were analysed using R 4.0.0. and Microsoft Excel 16.56. The completed questionnaires were cleaned and information verified. Based on the nature of the research questions, descriptive and exploratory approaches were used to delineate and describe the existence and use of edible insects in various Territories.

## Results and discussion

### Commonly consumed edible insects in selected Territories of South-Kivu

**Figure 2:**
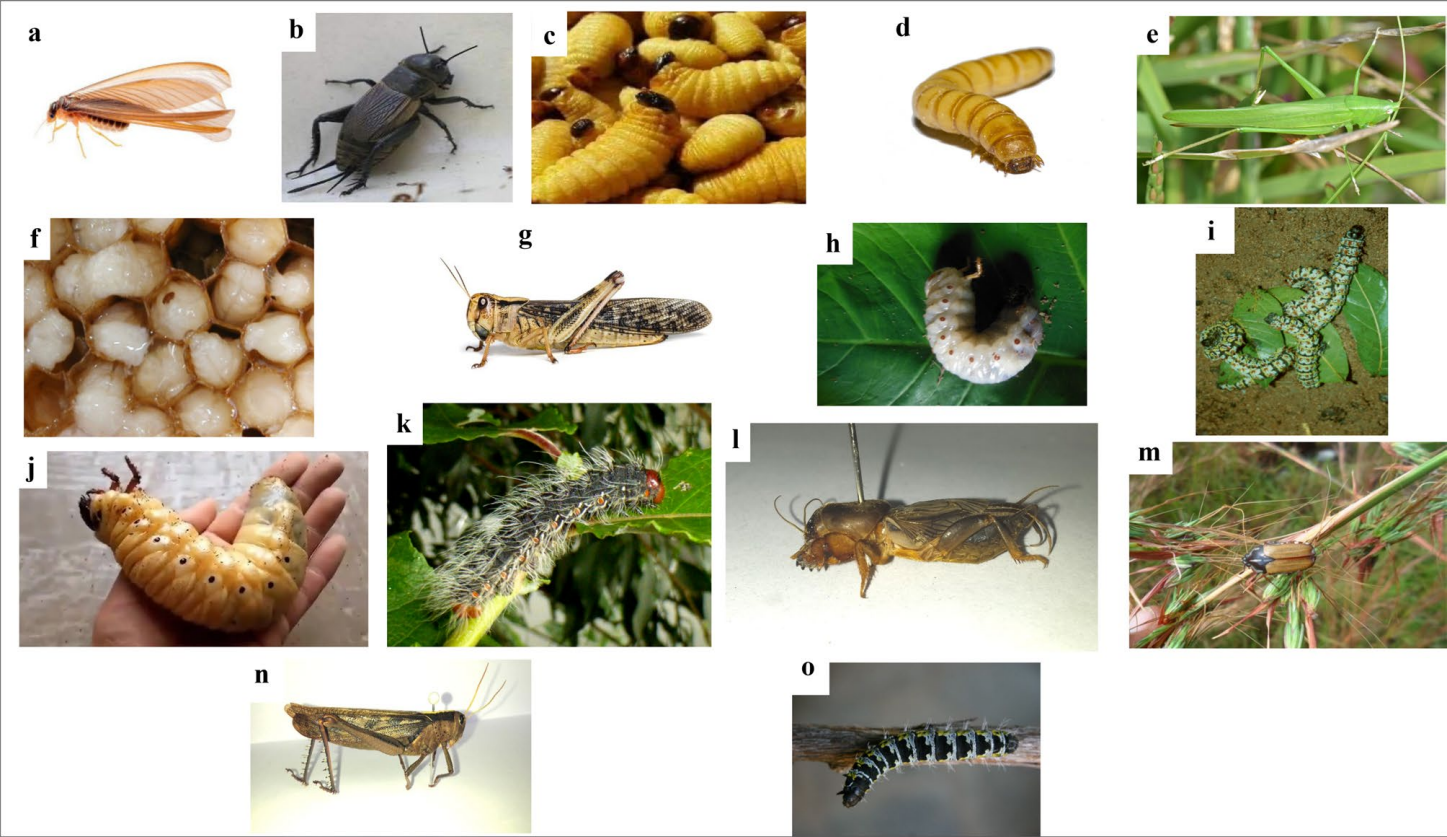
(**a**) *Macrotermes subhyalinus* (Termite); (**b**) *Acheta domesticus* (House cricket); (**c**) *Rhyncophorus phoenicis* larvae (Palm weevil larvae); (**d**) *Alphitobius diaperinus* larvae (Beetle); (**e**) *Ruspolia differens* (Grasshopper); (**f**) *Apis mellifera* larvae (Honey bee); (**g**) *Locusta migratoria* (Migratory locust); (**h**) *Rhynchophorus ferrugineus* larvae (Red palm weevil); (**i**) *Imbrasia oyemensis* (Caterpillar); (**j**) *Oryctes monoceros* larvae (Rhinoceros beetle); (**k**) *Imbrasia epimethea* (Caterpillar); (**l**) *Gryllotalpa Africana* (Mole cricket); (**m**) *Gnathocera trivittata* (Nsike); (**n**) *Nomadacris septemfasciata* (Red locust; (**o**) *Cirina forda* (Misigi). (Images mixed using Microsoft PowerPoint for Mac 16.29.1 https://www.microsoft.com).

A total of twenty-three edible insects including *Macrotermes subhyalinus*, *Acheta domesticus*, *Rhyncophorus phoenicis*, *Alphitobius diaperinus*, *Ruspolia differens*, *Gryllotalpa africana*, *Apis mellifera* larvae, *Nomadacris septemfasciata*, *Locusta migratoria*, *Rhynchophorus ferrugineus*, *Imbrasia oyemensis*, *Imbrasia epimethea*, *Oryctes monoceros*, *Cirina forda*, Nsike*,* Kigelegele*,* Kansenda*,* Bangwangwa*,* Maguina*,* Mingungu*,* Ngohangoha*,* Bikolongo and Bachache were inventoried as a source of food in Fizi, Kabare, Mwenga and Walungu Territories (Table 2, Fig. 2), belonging to nine families including Termitidae, Gryllidae, Curculionidae, Tenebrionidae, Acrididae, Gryllotalpidae, Apidae, Saturniidae and Scarabaeidae and five orders including Isoptera, Orthoptera, Coleoptera, Hymenoptera and Lepidoptera.

**Table 2:** Commonly consumed edible insects in selected Territories of South-Kivu.

Common name	Scientific name	Family	Order	Territory				Local name	Stage of consumption
				Fizi	Kabare	Mwenga	Walungu		
Termite	*Macrotermes subhyalinus*	Termitidae	Isoptera	+	+	+	+	Lolongue/Bushungwe	Winged adult
House cricket	*Acheta domesticus*	Gryllidae	Orthoptera	+	+	+	+	Makelele/Njanjala /hungwe/Ntoro	Adult
Palm weevil	*Rhyncophorus phoenicis*	Curculionidae	Coleoptera	+	−	+	−	Ebungu/Sololo/Mpose	Larvae
Beetle	*Oryctes rhinoceros*	Scarabaeidae	Coleoptera	+	−	+	−	Sungunya/Njukisha	Larvae and adult
Grasshopper	*Ruspolia differens*	Acrididae	Orthoptera	−	+	−	+	Minunu	Adult
Mole cricket	*Gryllotalpa Africana*	Gryllotalpidae	Orthoptera	−	+	−	+	Nkwananzi	Adult
Honey bee	*Apis mellifera*	Apidae	Hymenoptera	−	+	−	+	Magusha/Manyagu	Egg, larvae and pupa
Red locust	*Nomadacris septemfasciata*	Acrididae	Orthoptera	−	+	−	+	Mundurha	Adult
Migratory locust	*Locusta migratoria*	Acrididae	Orthoptera	−	+	−	+	Tondé	Adult
Red palm weevil	*Rhynchophorus ferrugineus*	Curculionidae	Coleoptera	−	+	+	−	Bivumbe	Larvae
Caterpillar	*Imbrasia oyemensis*	Saturniidae	Lepidoptera	−	+	+	−	Milanga	Larvae
Caterpillar	*Imbrasia epimethea*	Saturniidae	Lepidoptera	−	+	+	−	Taku/Tukumombo	Larvae
Rhinoceros beetle	*Oryctes monoceros*	Scarabaeidae	Coleoptera	−	−	+	−	Batumbu	Larvae
**Misigi**	*Cirina forda*	Saturniidae	Lepidoptera	−	−	+	−	Misigi	Larvae
**Kansenda**	NI	Curculionidae	Coleoptera	−	−	+	−	Kansenda	Larvae
**Nsike**	*Gnathocera trivittata*	Scarabaeidae	Coleoptera	−	+	−	+	Nsike	Adult
**Kigelegele**	NI	NI	NI	−	−	+	−	Kigelegele	Adult
**Bangwangwa**	NI	NI	NI	−	−	+	−	Bangwangwa	Adult
**Maguina**	NI	NI	NI	−	−	+	−	Maguina	Larvae
**Mingungu**	NI	NI	NI	−	−	+	−	Mingungu	Larvae
**Ngohangoha**	NI	NI	NI	−	−	+	−	Ngohangoha	Larvae
**Bikolongo**	NI	NI	NI	−	−	+	−	Bikolongo	Larvae
**Bachache**	NI	NI	NI	−	−	+	−	Bachache	Larvae

+ Insects present and consumed, − Insects not present, *NI* not identified. All of these edible insects are identified by local names, mainly in the dialects Kibembe (Fizi), Kirega (Mwenga) and Mashi (Kabare and Walungu) that are attached to specific physical characteristics or uses.

Among the inventoried edible insects, four (*M. subhyalinus*, *A. domesticus*, *R. phoenicis* and *A. diaperinus*) were found in Fizi, eleven (*M. subhyalinus*, *A. domesticus*, *R. differens*, *G. africana*, *A. mellifera* larvae, *N. septemfasciata*, *L. migratoria*, *R. ferrugineus*, *I. oyemensis*, *I. epimethea* and Nsike) in Kabare, seventeen (*M. subhyalinus*, *A. domesticus*, *R. phoenicis*, *A. diaperinus*, *R. ferrugineus*, *I. oyemensis*, *I. epimethea*, *O. monoceros*, *C. forda*, Kigelegele*,* Kansenda*,* Bangwangwa*,* Maguina*,* Mingungu*,* Ngohangoha*,* Bikolongo and Bachache) in Mwenga, and eight (*M. subhyalinus*, *A. domesticus*, *R. differens*, *G. africana*, *A. mellifera* larvae, *N. septemfasciata*, *L. migratoria*, and Nsike) in Walungu.

The *M. subhyalinus* and *A. domesticus* were used as sources of food in the whole study area, namely Fizi, Kabare, Mwenga and Walungu Territories, while *R. phoenicis* and *A. diaperinus* are consumed only in Fizi and Mwenga. In Kabare and Walungu Territories, *R. differens*, *G. africana*, *A. mellifera* larvae, *N. septemfasciata*, *L. migratoria* and Nsike are used as sources of food. Moreover, *R. ferrugineus*, *I. oyemensis* and *I. epimethea* are used as food sources in Kabare and Mwenga. However, *O. monoceros*, *C. forda*, Kigelegele*,* Kansenda*,* Bangwangwa*,* Maguina*,* Mingungu*,* Ngohangoha*,* Bikolongo and Bachache are only used as sources of food only in Mwenga Territory.

As for the stage of consumption, some edible insect species were consumed at the larval stage, including *R. phoenicis*, *A. diaperinus*, *A. mellifera* larvae, *R. ferrugineus*, *I. oyemensis*, *I. epimethea*, *O. monoceros*, *C. forda*, Maguina*,* Mingungu, Ngohangoha*,* Bikolongo, Bachach*e* and Kansenda, and others at the adult stage (*M. subhyalinus*, *A. domesticus*, *R. differens*, *G. africana*, Nsike*,* Kigelegele*,* and Bangwangwa). Unlike the other edible insects, the egg and pupa of *A. mellifera* are also consumed.

The wide biodiversity of edible insect species revealed in South Kivu depicts the importance of entomophagy in the region. Our findings largely agree with that of Bomolo et al.^31^. They reported a list of eleven edible insect species belonging to four families in Haut-Katanga Province, confirming that the Democratic Republic of Congo has a high diversity of edible insects, making it one of the most important biological diversity in Africa. This biodiversity in terms of edible insects in DRC was also confirmed by Raheem et al.^24^, who reported on traditional consumption and rearing of edible insects in Africa, Asia and Europe. Similarly, Kelemu et al.^16^ noted that most edible insects consumed in DRC belong to the orders reported in our findings.

This diversity could be associated with the richness of the natural environment conditions^33^ in each Territory as most edible insects are gathered from the wild^34^. In addition, the geographic distribution of host plants influences the availability of certain edible insects. A low number of caterpillar species has been attributed to marked deforestation, forest degradation and pollution^35,36^. This situation will likely worsen with the growing human populations and declining forest base^37^. Previous studies reported that edible insect’s consumption and preference are also influenced by their availability and cultures^38,39^.

Findings from this study line with Raheem et al.^24^, who reported that more than a thousand insect species are worldwide consumed at some stage of their life cycle. In addition, Lepidoptera consumed as caterpillars and Hymenoptera are mostly eaten in their larval or pupal stages like the *A. mellifera* mentioned from the survey to be consumed as egg and pupa. Adults and larvae of Coleoptera are consumed, while the Orthoptera, Isoptera and Hemiptera orders are mostly consumed as mature adults^16^. Moreover, Kulma et al.^40^ investigated the effect of developmental stage on the nutritional value of edible insects (*Blaberus craniifer* and *Zophobas morio*) reported no significant variations in basic nutrient content in protein quality expressed as the essential amino acid index. In contrast, they reported a significant difference in protein digestibility, fat content and lipid quality.

### Consumer preference for edible insects

**Figure 3:**
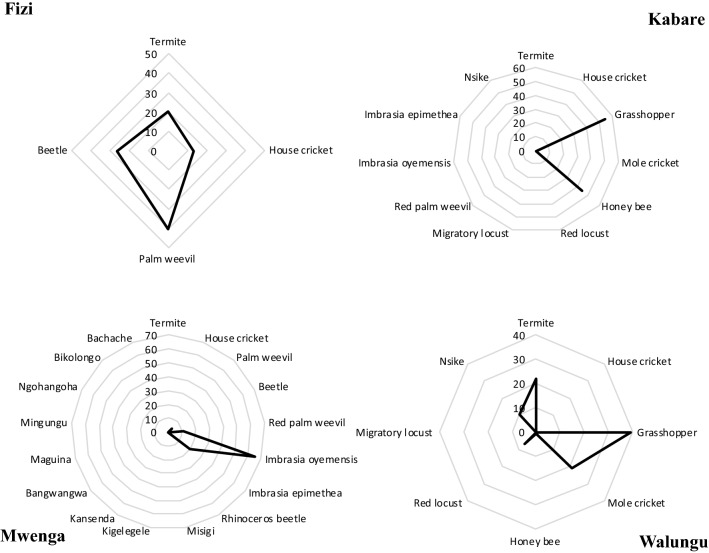
Most preferred edible insects in each Territory (n = 130). This is the number of times each edible insect is preferred. The preference is expressed in percentage. (Figures plotted using Microsoft Excel for Mac 16.56 and mixed using Microsoft PowerPoint for Mac 16.29.1 https://www.microsoft.com).

The inventoried edible insects were appreciated differently (Fig. 3). In Fizi, the most preferred edible insects were the *R. phoenicis* (41%), *A. diaperinus* (26%), *M. subhyalinus* (20%), and *A. domesticus* (13%), while in Kabare, the most preferred were *R. differens* (55%) and *A. mellifera* larvae (45%). Furthermore, *I. oyemensis* (65%), *I. epimethea* (20%), *R. ferrugineus* (11%) and *R. phoenicis* (4%) were the most preferred in Mwenga, while*R. differens* (39%), *M. subhyalinus* (22%), *G. africana* (21%), *Nsike* (10%), *N. septemfasciata* (7%) and *A. mellifera* larvae (1%) were most preferred in Walungu. Some edible insects were preferred in more than one Territory, such as *M. subhyalinus* (Fizi and Walungu), *R. phoenicis* (Fizi and Mwenga), *R. differens* and *A. mellifera* larvae (Kabare and Walungu).

Familiarity appears to be the key driving force, allowing most respondents to react positively to all edible species in terms of their willingness to eat them and thus address food and nutrition insecurity and related issues. This preference has been reported to be linked to availability^13,41^, ethnicity/cultures^42^, palatability^14^ and seasonality^43^, as some species are more prevalent and familiar in some Territories than others. In addition, indigenous knowledge and processing can also influence the preference of edible insect species^41^.

Bomolo et al.^31^ revealed that caterpillar consumption is common among ethnic populations in D.R. Congo. This is not the case for Walungu, Kabare and Fizi, although it is the most appreciated edible insects in Mwenga. In Katanga, the Bemba and Lamba tribes have a long history of caterpillar consumption since the eighteenth and nineteenth centuries^44^, while the other tribes (Musanga, Tetela, Mongo, Baluba, Rund, Bacongo, Katshokwe, Emba, Songe, Ndembo, Kaminungu, Kalwena, Kete, Basankusu, Kanyoka, Sanga, Mbote, Yombe, etc.) appear to lack a strong history of caterpillar consumption. In addition, some of these tribes are reluctant to engage in entomophagy, specifically to consume caterpillars due to their religious beliefs^45^. Studies have shown that education would play a crucial role in increasing the positive attitude towards edible insects among consumers^13,46^.

### Preference for edible insects

**Figure 4:**
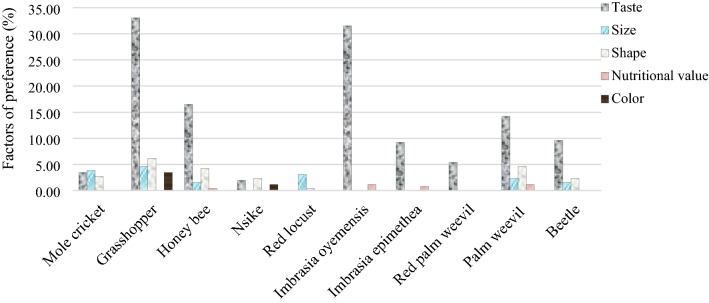
Preference of edible insects represented in two Territories. Respondents gave reasons for preferring one edible insect over another. The preference was based on taste, size, shape, nutritional value and colour. (Figures plotted using Microsoft Excel for Mac 16.56 https://www.microsoft.com).

The plotted data (Fig. 4) shows the appreciation factors for the most preferred inventoried edible insects (*G. africana*, *R. differens*, *A. mellifera* larvae, Nsike, *N. septemfasciata* and *I. oyemensis*) represented in two Territories (n = 260). Respondents rated them according to taste, size, shape, nutritional value and colour. Most of these insects were valued for their taste, especially *R. differens* (33%), *I. oyemensis* (32%), *A. mellifera* larvae (17%), *G. africana* (3%), and Nsike (2%), except for *N. septemfasciata,* which was valued for its size (3%) and shape (0.4%). As for size, *R. differens* (5%), *G. africana* (4%) and *N. septemfasciata* (3%) were the most valued. Most of these edible insects were not valued for their nutritional value or colour except for *A. mellifera* larvae (0.4%) and *I. oyemensis* (1%) for nutritional value and *R. differens* (3%) and Nsike (1%) for colour.

**Figure 5:**
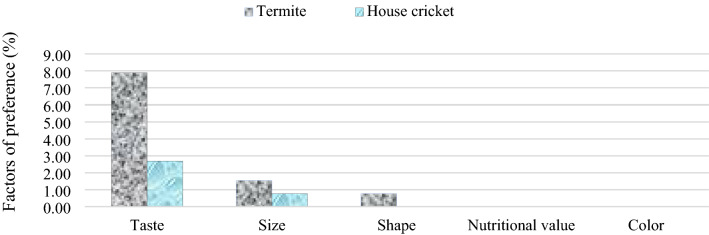
Preference of Termites and House crickets in the study area. Respondents gave reasons for preferring one edible insect over another. The preference was based on taste, size, shape, nutritional value and colour. (Figures were plotted using Microsoft Excel for Mac 16.56 https://www.microsoft.com).

Considering *M. subhyalinus* and *A. domesticus* represented in all Territories (n = 520). Irrespective of Territories, their appreciation depended only on their taste, size and shape (Fig. 5). More appreciation was based on the taste with 8% and 3% against 2% and 1% for the size for *M. subhyalinus* and *A. domesticus,* respectively; only *M. subhyalinus* was appreciated for its shape (1%).

Entomophagy habits differ from country to country and culture to culture, as do preference factors. Insect consumption depends not only on sensory characteristics^13^ and nutritional value^10,41^ but also on customs, ethnic preferences, prohibitions^7^, and medicinal properties^47^. Insects were once associated with filth, fear of contamination and disease, as well as psychological and biased thinking about taste, smell, and colour^48^, with a sense of disgust that entomophagy was motivated by starvation and is merely a survival mechanism^13^. This is far from the truth, as insects are not inferior to other protein sources, such as fish, chicken, and beef. However, it will take a bit more motivation to reverse this mentality^49^. It is possible to explore edible insects for consumption and increase the possibility of replacing animal products with insects, given that there is evidence that they are clean, tasty, and nutritious^50^.

In addition, insects have too many ecological advantages over other animal protein sources^6^. Some studies in European countries such as the Netherlands^51^ on the acceptance of entomophagy have shown that people who have eaten insects in the past show significantly more positive attitudes towards entomophagy than people who have not and are more likely to eat them again. Therefore, it seems important to encourage people to take the "first step" and familiarize them with insect consumption. Therefore, consumer "education" about entomophagy should be practiced in its broadest sense^51^.

### Seasonal availability of various inventoried edible insects

Regardless of Territory, three edible insect groups, mainly *A. domesticus*, *A. diaperinus* and *A. mellifera* larvae, are abundant throughout the year (Table 3). *Nomadacris septemfasciata* is also available throughout the year but abundant only during 5 months and less abundant from February to August. *Gryllotalpa africana* and *R. ferrugineus*, on the other hand, were abundant throughout the rainy season and are less abundant in the dry season. *G. africana* and *R. ferrugineus* are not abundant, respectively, in August and June. Both species are all unavailable in July. Elsewhere, *M. subhyalinus*, *R. phoenicis* and *R. differens* are available half the year (6 months). However, *M. subhyalinus* and *R. differens* were less available for 3 months of the year while *R. phoenicis* are only available for one month. Other groups such as *Oryctes monoceros*, *Cirina forda*, *Ngohangoha*, *I. oyemensis*, Nsike*,* Mingungu*,* Bikolongo and Bachache were abundant for only 3 to 4 months of the year. In contrast, *I. epimethea*, Maguina*,* Kigelegele*,* Kansenda and Bangwangwa were the most available and abundant for only one to two months of the year.

**Table 3:** Seasonal availability of various inventoried edible insects.

Insect species	Rain season					Dry season			Rain season					
	Jan	Feb	Mar	Apr	May	Jun	Jul	Aug	Sep	Oct	Nov	Dec	TMA	TLA
Termite	+	+	+	+	−	0	0	0	−	−	+	+	6	3
House cricket	+	+	+	+	+	+	+	+	+	+	+	+	12	0
Palm weevil	0	0	0	+	+	+	+	−	0	0	+	+	6	1
Beetle	+	+	+	+	+	+	+	+	+	+	+	+	12	0
Grasshopper	+	−	−	+	+	0	0	0	−	+	+	+	6	3
Mole cricket	+	+	+	+	+	0	0	−	+	+	+	+	9	1
Honey bee	+	+	+	+	+	+	+	+	+	+	+	+	12	0
Red locust	+	−	−	−	−	−	−	−	+	+	+	+	5	7
Migratory locust	−	−	0	0	0	0	0	0	0	0	+	+	2	2
Red palm weevil	+	+	+	+	+	−	0	0	+	+	+	+	9	1
*Imbrasia oyemensis*	0	+	+	+	−	0	0	0	0	0	0	0	3	1
*Imbrasia epimethea*	0	+	+	−	0	0	0	0	0	0	0	0	2	1
Rhinoceros beetle	+	+	+	−	−	0	0	0	0	0	0	+	4	2
**Misigi**	+	+	+	−	0	0	0	0	0	0	0	+	4	1
**Kansenda**	0	0	+	−	0	0	0	0	0	0	0	0	1	1
**Nsike**	0	0	0	+	+	+	−	−	0	0	0	0	3	2
**Kigelegele**	0	+	−	0	0	0	0	0	0	0	0	0	1	1
**Bangwangwa**	−	0	0	0	0	0	0	0	0	0	0	+	1	1
**Maguina**	0	+	+	−	0	0	0	0	0	0	0	0	2	1
**Mingungu**	0	0	+	+	+	−	0	0	0	0	0	0	3	1
**Ngohangoha**	0	0	0	0	0	0	+	+	+	+	−	0	4	1
**Bikolongo**	0	0	+	+	+	−	0	0	0	0	0	0	3	1
**Bachache**	0	+	+	+	−	0	0	0	0	0	0	0	3	1

+ month of availability, − month of less availability, 0 month of none availability, *TMA* Total month of availability, *TLA* Total month of less availability.

Availability is an important factor in the consumption of edible insects^14^. Our results corroborate of those of Ebenebe et al.^20^, who pointed out that most of the harvesting is done during the rainy season in Nigeria, especially for winged termites, cricket, caterpillars, *A. domesticus*, *G. africana*, and greenish beetle. Smith and Paucar^52^ suggested that vibrations caused by rain and the sound of thunder would trigger their emergence. Chakravorty et al.^14^ also confirmed that the availability of edible insects is seasonal, stating that peak numbers of edible beetles occur from June to September before decreasing in winter and early spring. They also reported that Odonata and Orthoptera were most abundant in September and October (late summer).

Insects of the order Hemiptera and Hymenoptera are less abundant from November to February (winter), while others such as bugs and ants are available throughout the year. This would be attributed to seasonal changes in various regions of the world. In the Central African Republic, the average consumption of caterpillars increases due to their greater abundance during the rainy season^13^, as seasonal availability and edible insect consumption are correlated^15^. In contrast to our findings, all developmental stages of *R. differens* can be found throughout the year in non-swarming populations but at low densities during dry seasons and high densities during rainy seasons^53^.

### Host plants for various inventoried edible insects

Some edible insects such as *M. subhyalinus*, *A. domesticus*, *G. africana*, *A. mellifera* larvae, Kigelegele and Bangwangwa do not necessarily have host plants. However, *R. phoenicis*, *A. diaperinus*, *R. differens*, *N. septemfasciata*, *L. migratoria*, *R. ferrugineus*, *I. oyemensis*, *I. epimethea*, *O. monoceros*, *C. forda*, Kansenda and Nsike require host plants to serve either for habitat or food source (Table 4). Edible insects such as *N. septemfasciata* and *L. migratoria* are dangerous as they use maize (*Zea mays*), rice (*Oryza sativa*), soybean (*Glycine max)*, sugar cane (*Saccharum officinarum*), groundnut (*Arachis hypogaea*) and sweet potato (*Ipomoea batatas*) crops as host plants, while the latter are also sources of staple foods for humans. On the other hand, other species *R. phoenicis*, *A. diaperinus*, *R. ferrugineus*, *O. monoceros* and Kansenda, were hosted on *Raffia palm*, *Cocos nucifera*, *Elaeis guineensis*, and *Mangifera spp* trees which are not only sources of food for humans and a source of income for many people. Their signs of presence differ from one to another. The presence of *R. phoenicis*, *A. diaperinus*, *R. ferrugineus*, *O. monoceros* and Kansenda is noticed by cracking noises in palm trunks, odour, and their activities at the hole of the entrance. Furthermore, caterpillar smells and typical bird songs were signs of presence for *I. oyemensis* and *I. epimethea*. Moreover, *G. africana*, *N. septemfasciata*, *L. migratoria* and *C. forda* are noticed by whistling and canals in the wet ground.

**Table 4:** Host plants of various consumed edible insects.

Insect species	Host plants		Signs of presence
	Common name	Scientific name	
Termite	NA	NA	NA
House cricket	NA	NA	NA
Palm weevil	Palm, coconut and African oil palm	*Raffia palm, Cocos nucifera* and *Elaeis guineensis*	Cracking noises in palm trunks and odour
Beetle	Palm, yellow flame and mango	*Raffia palm, Peltophorum pterocarpum* and *Mangifera spp*	Indication of its activity at the hole of entrance and cracking noises in the palm
Grasshopper	Grass, guinea grass and giant rat's tail grass	*Digitaria sp, Panicum maximum,* and *Sporobolus pyramidalis*	NA
Mole cricket	NA	NA	Whistling and canals in the wet ground
Honey bee	NA	NA	NA
Red locust	Maize, rice, soybean, sugarcane, groundnut and sweet potato	*Zea mays, Oryza sativa, Glycine max, Saccharum officinarum, Arachis hypogaea* and *Ipomoea batatas*	Whistling
Migratory locust	Maize, rice, soybean, sugarcane, groundnut and sweet potato	*Zea mays, Oryza sativa, Glycine max, Saccharum officinarum, Arachis hypogaea* and *Ipomoea batatas*	Whistling
Red palm weevil	Coconut, African oil palm and sugarcane	*Cocos nucifera, Elaeis guineensis* and *Saccharum officinarum,*	Indication of its activity at the entrance hole, odour and cracking noises in the palm
*Imbrasia oyemensis*	Red mangrove	*Rhizophora mangle*	Caterpillar smells and typical bird songs
*Imbrasia epimethea*	Red mangrove and African blackwood	*Rhizophora mangle* and *Erythrophleum africanum*	Caterpillar smells and typical bird songs
Rhinoceros beetle	Coconut and African oil palm	*Cocos nucifera* and *Elaeis guineensis*	Indication of its activity at the hole of entrance and Cracking noises in the palm
**Misigi**	Red mangrove and African blackwood	*Rhizophora mangle* and *Erythrophleum africanum*	Whistling
**Kansenda**	Palm and coconut	*Raffia palm* and *Cocos nucifera*	Cracking noises in palm trunks and odour
**Nsike**	Jaragua grass, Weeping lovegrass and Giant rat's tail grass	*Hyparrhenia rufa, Eragrostis curvula* and *Sporobolus pyramidalis*	NA
**Kigelegele**	NA	NA	NA
**Bangwangwa**	NA	NA	NA
**Maguina**	NYD	NYD	NYD
**Mingungu**	NYD	NYD	NYD
**Ngohangoha**	NYD	NYD	NYD
**Bikolongo**	NYD	NYD	NYD
**Bachache**	NYD	NYD	NYD

*NA* Not applicable, *NYD* Not yet determined.

Ebenebe and collaborator^20^ highlighted that certain edible insects are associated with the following host plants: cricket-yam; yam beetle-yam; African palm weevil-raffia palm; Rhinoceros beetle-raffia palm, oil palm, coconut tree; butterfly-iroko (*Chlorophora excelsa*), locust bean seed (*Parkia biglobosa*), flamboyant tree (*Delonix regia*), croton (*Croton tiglium*) and ngwu tree; grasshopper and honey bee-*Jatropha gossyplifolia*, *Citrus sinensis*, *Morinda lucida*, *Psidium guajava* and *Sarcocepha laifolius*. According to Ngute et al.^54^, five of the eleven caterpillar species studied in ﻿central Cameroon were reported to have only one host plant, while others had more than one. They identified eighteen plants, of which eleven are restricted to natural forest habitats, including *Entandrophragma cylindricum* and *Baillonella toxisperma*. Although many of the identified caterpillar host plants are generally wild, a few are domesticated and grown in home gardens and agroforestry systems such as *Mangifera indica* and *Dacryodes edulis*, or are in the process of domestication such as *Ricinodendron heudelotii*, *B. toxisperma* and *E. cylindricum*^54^. Also, it should be noted that most of the hosts are plants used as a source of food and revenue; for example, *B. toxisperma* is a class A timber species, which produces fruits with a highly valued and edible oil is extracted^55^.

It has been reported that out of 21,252 observations, *R. differens* were observed 20,915 (98%) times on grasses and sedges, with a total of 19 grass species (*Poaceae*) and two sedge species (*Cyperaceae*). Among the grasses the dominant species were *P. maximum*, *B. ruziziensis*, *C. gayana*, *H. rufa*, *Cynodon dactylon*, *Sporobolus pyramidalis* and *P. purpureum*^53^. When reared in the laboratory, *R. differens* accept artificial food, leaves, flowers, and grains of many types of grass, including cultivated cereals^56,57^. At this point, our findings line with those of Meutchieye et al.^19^, who observed that the cracking noises in the palm trunks three to four weeks after the final collection of palm wine, the yellow of the internal raffia bamboo, caterpillar odours and typical bird songs and whistling are the signs indicating the presence of *R. phoenicis*, caterpillars, *R. differens* and field crickets.

### Harvesting and processing techniques of edible insects

Thistudyfound that harvesting time and techniques vary according to local knowledge, practices, and insect species (Table 5). Three harvesting techniques were identified, including trapping, collection. *M. subhyalinus* (during and after the first rains) and *R. differens* (during the swarming season) are trapped with light near a container; once attracted by light, they fall inside before being collected then de-winged. In addition, *R. differens* are also hand-picked on host plants while *A. domesticus* are collected by trapping and handpicking at any time. Furthermore, *R. phoenicis*, *A. diaperinus*, *G. africana*, *A. mellifera* larvae, *N. septemfasciata*, *L. migratoria*, *R. ferrugineus*, *I. oyemensis*, *I. epimethea*, *O. monoceros*, *C. forda*, Nsike, Maguina, Mingungu, Ngohangoha, Bikolongo and Bachache are hand-picked.

**Table 5:** Harvesting and processing techniques for various consumed edible insects in selected Territories.

Insect species	Harvesting techniques	Harvesting period	Processing methods	Preservation techniques
Termite	Light trapping near a container. Termites attracted by light fall inside before being collected and have their wing removed	During and after the first rains	De-winged, roasted or dry-fried; also eaten raw	Drying
House cricket	Trapping and handpicking	Any time	De-winged, roasted or dry-fried	Drying
Palm weevil	Handpicking after signs of their presence is detected	Any time based on signs	Gut removed, boiled, fried or roasted, sometimes prepared in stews	Drying
Beetle	Handpicking after signs of their presence is detected	Any time based on signs	Washed, boiled, fried or roasted, sometimes prepared in stews	Drying
Grasshopper	During the swarming season, the light trapping technique is used to attract grasshoppers in addition to handpicking on host plants	Soon after the dark for light trapping and morning before the sun rises when they could not fly for handpicking	De-winged, roasted or dry-fried	Dry-fried during the swarming season
Mole cricket	Handpicking	In the evening, following their small holes	De-winged, roasted or dry-fried and boiled	Drying
Honey bee	Collecting honeycomb from the hive following with honey extraction	At night preferably	Boiled	None
Red locust	Handpicking on host plants	Morning time	De-winged, roasted or dry-fried	Drying
Migratory locust	Handpicking on host plants	Morning time	De-winged, roasted or dry-fried	Drying when it is enough
Red palm weevil	Handpicking after signs of their presence is detected	Any time based on signs	Washed, boiled, fried or roasted, sometimes prepared in stews	Drying
Caterpillar	Handpicking: caterpillar directly picked after signs of their presence are detected	Any time, preferably morning and evening hours	Boiled, fried or roasted, sometimes prepared in stews	Drying
Rhinoceros beetle	Handpicking after signs of their presence are detected	Any time, preferably morning and evening hours	Boiled, fried or roasted, sometimes prepared in stews	Drying
**Misigi**	Handpicking after signs of their presence are detected	Any time, preferably morning and evening hours	Boiled, fried or roasted, sometimes prepared in stews	Drying
**Kansenda**	Handpicking after signs of their presence are detected	Any time, preferably morning and evening hours	Fried	Drying
**Nsike**	Handpicking on host plants	During the sunny period, they are easy to identify and handpicking on the top of the host plants	De-winged, roasted or dry-fried	Dry-fried during the swarming season
**Kigelegele**	Handpicking after signs of their presence are detected	Any time, preferably morning and evening hours	Boiled and Fried	Drying
**Bangwangwa**	Handpicking after signs of their presence are detected	Any time, preferably morning and evening hours	Boiled and roasted	Drying
**Maguina**	Handpicking after signs of their presence are detected	Any time, preferably morning and evening hours	Boiled, fried or roasted, sometimes prepared in stews	Drying
**Mingungu**	Handpicking after signs of their presence are detected	Any time, preferably morning and evening hours	Boiled and roasted	Drying
**Ngohangoha**	Handpicking after signs of their presence are detected	Any time, preferably morning and evening hours	Boiled, fried or roasted, sometimes prepared in stews	Drying
**Bikolongo**	Handpicking after signs of their presence are detected	Any time, preferably morning and evening hours	Boiled and roasted	Drying
**Bachache**	Handpicking after signs of their presence are detected	Any time, preferably morning and evening hours	Boiled, fried or roasted, sometimes prepared in stews	Drying

Some edible insects are collected only in the morning or evening, while others are collected at any time. The harvest decreases with the intensity of the sun. *Ruspolia differens* are light trapped soon after the dark. Like *N. septemfasciata*, *L. migratoria* and Nsike, *R. differens* are also hand-picked in the morning before the sun rises when they cannot fly. On the other hand, *A. mellifera* larvae and *G. africana* are collected at night and in the evening, preferably. *Rhyncophorus phoenicis*, *A. diaperinus*, *R. ferrugineus*, *I. oyemensis*, *I. epimethea*, *O. monoceros*, *C. forda*, Maguina, Mingungu, Ngohangoha, Bikolongo and Bachache are collected Any time during the day, but preferably in the morning and evening hours.

Processing methods and preservation techniques vary according to the type of edible insect and the purpose, whether for direct consumption or preservation. Generally, they are all dried or dry-fried for preservation except for the honeybee (Table 5). Most edible insects with wings such as *M. subhyalinus*, *A. domesticus*, *R. differens*, *G. africana*, *N. septemfasciata*, *L. migratoria* and *Nsike* are de-winged before being roasted, dry-fried, fried or boiled for *G. africana*. *M. subhyalinus* are also eaten raw. *Rhyncophorus phoenicis*, *A. diaperinus*, *R. ferrugineus*, *I. oyemensis*, *I. epimethea*, *O. monoceros*, *C. forda*, Maguina, Ngohangoha and Bachache are gut removed, washed, boiled, fried or roasted, and sometimes prepared in stews. *A. mellifera* larvae are boiled while Kigelegele, Bangwangwa, Mingungu, and Bikolongo are boiled and roasted.

These results are consistent with those of Meutchieye et al.^19^, as well as those of Ebenebe et al.^20^, who also pointed out that hand picking is one of the major techniques used to harvest crickets, Rhinoceros beetle, stinking grasshopper (in the early hours of the morning), African palm weevil (from rotting palm tree) and caterpillars. In contrast, light trapping is used to harvest termites, green grasshoppers, house cricket and mole cricket. In addition, Ebenebe et al.^20^ added that crickets were also harvested by digging out from the tunnels during the dry season and that house cricket was also harvested by digging out from small sound mounds with depression that indicates their entrance. In contrast to the results of this study, Ebenebe et al.^20^ stated that mole crickets were harvested by light trapping. Singing a "Wee wee" song at the base of the host tree is another notable technique used to harvest the caterpillar of a particular moth species. The more you sing, the more they fall from the top of the tree and are harvested.

To harvest enough *R. differens* by light trapping during the swarming season, locals lit the lamps/bulbs outside the houses and secure the areas where these lamps/bulbs are located with corrugated iron sheets bent into a cone shape leading to baskets, small drums where the falling *R. differens* are harvested^58^. For non-swarming *R. Differens*, harvesting is done early in the morning between 6 and 7 AM, when they are inactive and unable to fly^17^. Dao et al.^59^ also reported two harvesting techniques: direct termite collections from their mounds and termite trapping in containers. The direct collection involves partially destroying termite mounds, while trapping implies looking for signs of termite presence such as mud sheets and tracks on the ground. Techniques depend on the termite genus. Small termite mounds of *Trinervitermes* and *Cubitermes* can be dug with a hoe or pickaxe, and the mounds are collected in the morning between 6 and 9 AM. The traps are placed between 6 and 8 AM or in the evening around 6 PM. They are covered with foliage or pieces of cloth to protect them from the sun. The same harvesting techniques were recently described in Ghana^60^.

It was reported that edible insects were sun-dried, baked, steamed and processed into crackers, muffins and sausage meat^61,62^. Our findings confirm those of Ebenebe et al.^20^, who also found that salted roasting is one of the techniques used to process termites, crickets, Rhinoceros beetle, grasshopper and locust. They added that grasshopper and locust are consumed dried as well. On the other hand, African palm weevils are consumed raw or fried with pepper sauce. This study found that drying was the most used preservation technique as it is the most widely used technology for increasing the shelf-life of foods. The drying ranges from traditional, including roasting, frying and solar drying, to modern freeze-drying and microwave-assisted drying^63^.

## Conclusion and recommendations

The wide biodiversity of edible insects observed depicts the importance of entomophagy in the region. Twenty-three edible insects were recorded belonging to nine families and five orders. These insects are consumed as larvae, adults or as eggs and pupa. About four of them were the most popular edible insects in Fizi and Mwenga, two in Kabare, and six in Walungu Territory. Additionally, *R. differens*, *I. oyemensis*, *A. mellifera* larvae, *G. africana* and Nsike were the most preferred for their taste. Besides, some are available throughout the year, while the others are less available. Many plants have been recorded as host plants, including plants used as food sources, feed and income for humans. Harvesting strategies and time, processing methods, and preservation techniques vary according to local knowledge and practices and insect species. These findings suggest similar studies in other provinces and further research on the nutritional and safety profiling of processed and non-processed edible insects while encouraging the rearing of certain edible insects for mass production as the demand is too high.
